# AMP Affects Intracellular Ca^2+^ Signaling, Migration, Cytokine Secretion and T Cell Priming Capacity of Dendritic Cells

**DOI:** 10.1371/journal.pone.0037560

**Published:** 2012-05-18

**Authors:** Elisabeth Panther, Thorsten Dürk, Davide Ferrari, Francesco Di Virgilio, Melanie Grimm, Stephan Sorichter, Sanja Cicko, Yared Herouy, Johannes Norgauer, Marco Idzko, Tobias Müller

**Affiliations:** 1 Department of Gastroenterology, University Medical Centre Freiburg, Freiburg, Germany; 2 Department of Pneumology, University Medical Centre Freiburg, Freiburg, Germany; 3 Section of General Pathology, Department of Experimental and Diagnostic Medicine, and Interdisciplinary Center for the Study of Inflammation (ICSI), University of Ferrara, Ferrara, Italy; 4 Department of Dermatology, University Medical Centre Freiburg, Freiburg, Germany; 5 Department of Dermatology, University Medical Centre Jena, Jena, Germany; University of Rochester Medical Center, United States of America

## Abstract

The nucleotide adenosine-5′-monophosphate (AMP) can be released by various cell types and has been shown to elicit different cellular responses. In the extracellular space AMP is dephosphorylated to the nucleoside adenosine which can then bind to adenosine receptors. However, it has been shown that AMP can also activate A_1_ and A_2a_ receptors directly. Here we show that AMP is a potent modulator of mouse and human dendritic cell (DC) function. AMP increased intracellular Ca^2+^ concentration in a time and dose dependent manner. Furthermore, AMP stimulated actin-polymerization in human DCs and induced migration of immature human and bone marrow derived mouse DCs, both via direct activation of A_1_ receptors. AMP strongly inhibited secretion of TNF-α and IL-12p70, while it enhanced production of IL-10 both via activation of A_2a_ receptors. Consequently, DCs matured in the presence of AMP and co-cultivated with naive CD4^+^CD45RA^+^ T cells inhibited IFN-γ production whereas secretion of IL-5 and IL-13 was up-regulated. An enhancement of Th2-driven immune response could also be observed when OVA-pulsed murine DCs were pretreated with AMP prior to co-culture with OVA-transgenic naïve OTII T cells. An effect due to the enzymatic degradation of AMP to adenosine could be ruled out, as AMP still elicited migration and changes in cytokine secretion in bone-marrow derived DCs generated from CD73-deficient animals and in human DCs pretreated with the ecto-nucleotidase inhibitor 5′-(alpha,beta-methylene) diphosphate (APCP). Finally, the influence of contaminating adenosine could be excluded, as AMP admixed with adenosine desaminase (ADA) was still able to influence DC function. In summary our data show that AMP when present during maturation is a potent regulator of dendritic cell function and point out the role for AMP in the pathogenesis of inflammatory disorders.

## Introduction

Different cell types such as activated platelets [1], neutrophils [2], and eosinophils [3] have been shown to release adenosine-5′-monophosphate (AMP). Furthermore hydrolysis of ATP or ADP by ecto-ATPases (CD39) leads to the accumulation of AMP in the extracellular space, whereas AMP itself can be degraded by ecto-5′-nucleotidase (CD73) to adenosine which is a well characterized signaling molecule binding to different adenosine receptor subtypes. The biological effects of AMP include bronchoconstriction [4], stimulation of DNA synthesis, and mitogenesis [5]. In the past, most of the effects elicited by AMP have been attributed to the fact that AMP can be degraded rapidly to adenosine. However, there is good evidence that AMP can also directly bind to A_1_ and A_2A_ receptors without being dephosphorylated to adenosine before [6], [7]. In contrast, GPR80 (GPR99) which has been claimed to be a receptor specific for AMP has turned out to be a receptor for citric acid cycle intermediates but not for AMP [8], [9].

Dendritic cells (DCs) are antigen presenting cells specialized in activating naive T cells thereby initiating primary immune responses [10], [11]. DCs originate from hematopoietic stem cells and migrate into target sites to capture antigens [11]. During circulation through the body DCs undergo maturation, a process that entails acquisition of high levels of surface MHC and co-stimulatory molecules, as well as the production of different cytokines and chemokines. In secondary lymphoid organs DCs play a crucial role in the development of Th1/Th2-driven immune responses through the release of cytokines and chemokines [11]. Additionally, they also produce several pro-inflammatory cytokines including TNF-α, IL-1β, IL-6, and IL-8 profoundly affecting the outcome of inflammatory reactions [12]. Hence DCs have been shown to be involved in the pathogenesis of inflammatory disorders such as bronchial asthma [13], [14], [15].

DCs express different purinergic receptors including adenosine receptors. Consequently extracellular nucleotides such as ATP, ADP, UTP, or UDP have been demonstrated to affect maturation, migration, cytokine secretion, and T-cell priming capacity of DCs [16], [17], [18], [19], [20]. However, little is known about the influence of AMP on dendritic cell function.

## Materials and Methods

### Ethics statement

The use of human blood samples was approved by the ethics committee at the University of Freiburg (Approval ID 03/10). Written consent was obtained from all participants.

All experiments involving animals were carried out in strict accordance with the national protection of Animals act. Animal experiments were approved by the local animal ethics committee (Regierungspräsidium Freiburg).

### Preparation of human dendritic cells

Peripheral mononuclear cells were isolated from heparin-anticoagulated blood of healthy volunteers using a Ficoll gradient. After separation, the leukocyte-containing pellet was resuspended in 2 ml of PBS containing 2 mM EDTA and 0.5% BSA. Cells were separated with anti-CD14 mAb-coated MicroBeads using Macs single use separation columns from Miltenyi Biotec (Bergisch Gladbach, Germany). The CD14^+^ cells were cultured for 5 days in RPMI 1640 medium containing 10% FCS, 1% glutamine, 50 IU/ml penicillin, 50 µg/ml streptomycin, 1,000 U/ml IL-4, and 10,000 U/ml GM-CSF (Natutec, Frankfurt, Germany) at 37°C in a humidified atmosphere with 5% CO_2_. Maturation of DCs was induced by 48 h incubation in the presence of 3 µg/ml LPS (Sigma-Aldrich, Germany).

### Intracellular Ca^2+^ measurements

Intracellular-free Ca^2+^ was measured in fura-2/AM-loaded DCs using the digital fluorescence microscope unit Attofluor (Zeiss, Oberkochen, Germany), as previously described [19]. Briefly, DCs were incubated with 2×10^−6^ M fura-2/AM for 30 min at 37°C in a Ca^2+^- and Mg^2+^-free buffer. Cells were then washed twice and resuspended in the same buffer containing 1.5 mM CaCl_2_ and MgCl_2_. Cells were stimulated with nucleotides and [Ca^2+^]_i_ changes determined with the 340/380 excitation ratio at an emission wavelength of 505 nm.

### Actin polymerization

The content of filamentous actin was analyzed by flow cytometry with NBD-phallacidin staining [19]. Briefly, aliquots (50 µL) of DCs suspensions (5×10^5^ cells/mL) were withdrawn at the indicated time intervals and fixed in a 7.4% formaldehyde buffer. After 1 h, cells were mixed with the staining cocktail containing 7.4% formaldehyde, 0.33×10^−6^ M NBD-phallacidin, and 1 mg/ml lysophosphatidylcholine. The mean channel number of the fluorescence intensity of each sample was measured by flow cytometry (FacsCalibur, BD, Franklin Lakes, USA). The relative f-actin content in comparison to the medium control was calculated.

### Mice

C57BL/6 mice, OVA-TCR transgenic OT-II mice on a C57/Bl6 mice background (6–8 week-old) were bred at the animal facilities at the University Hospital Freiburg. CD73-deficient mice (CD73−/−) on C57BL/6 background were kindly provided by Linda Thompson and backcrossed with our C57BL/6 strains. All experiments were performed according to institutional guidelines of the animal ethics committee from the German government.

### Generation of bone marrow-derived DCs (BMDCs)

DCs were prepared as previously described [21], [22]. Briefly, bone marrow cells from wt and CD73-deficient (CD73−/−) mice (both on C57BL/6 background) were grown in RPMI 1640 medium supplemented with gentamycin, 2-mercaptoethanol, 10% FCS (Biocell Laboratories), and recombinant murine GM-CSF (200 IU/ml). On days 3, 6, and 8 the medium was changed and GM-CSF was added. On day 9 cells were pulsed overnight with 100 µg/ml LPS-low OVA (MW 45 kDa/Worthington Biochemicals, Lakewood, USA) or vehicle. In some experiments cells were stimulated with different concentrations of AMP, adenosine, or adenosine desaminase 30 min prior to overnight pulsing. The next day supernatants for cytokine analysis were harvested and non-adherent DCs were collected and washed to remove free OVA. The purity of bone marrow–derived DCs was greater than 90% as determined by CD11c staining.

### Migration assay

Experiments were performed in triplicate using 24-well transwell chambers with a pore size of 5 µM (Nunclon, Langenselbold, Germany) [21], [22]. Buffer or different concentrations of AMP were added into the lower compartment wells. Human or murine cells (10^5^ cells/well) were added to the upper compartment and incubated at 37°C for 90 min in a humidified atmosphere. Migrated DCs in the lower chamber were stained with trypan blue and counted in a hematocytometer. Results are shown as chemotatic index, calculated as the number of cells in the lower chamber containing the different stimuli divided by the number of cells in the chamber containing medium alone.

### Cytokine measurements

To measure cytokine levels in the supernatants of DCs, human or murine cells (2×10^5^/well) were stimulated with the indicated concentrations of AMP or adenosine (purchased from Sigma, Deisenhofen, Germany) 1 hour or 30 min prior to overnight pulsing with LPS (3 µg/ml) or OVA. The next day cell-free supernatants were harvested and the presence of IL-10, IL-12 and TNF-α was assessed by ELISA (R&D systems, Minneapolis, USA).

### T-cell differentiation assay

Peripheral mononuclear cells were separated from buffy coats using a Ficoll (GE Healthcare, Uppsala, Sweden) gradient. After separation, the leukocyte-containing pellet was resuspended in 2 ml of PBS containing 2 mM EDTA and 0.5% BSA. Cells were separated with CD4^+^ T-cell isolation kit (Miltenyi, Bergisch Gladbach, Germany). CD4^+^ T-cells were depleted of CD45RO^+^ cells with CD45RO microbeads (Miltenyi, Bergisch Gladbach, Germany). 5×10^5^ CD4^+^CD45RA^+^ cells were co-cultured with 1×10^5^ DCs for 5 days. After 5 days cells were re-stimulated with 10 ng/mL phorbol myristate acetate (PMA) and 1 µg/mL ionomycin (Sigma-Aldrich, Taufkirchen, Germany). After additional 2 days supernatants were collected and levels of IL-5, IL-13, and IFN-γ were analyzed by ELISA (R&D systems, Minneapolis, USA).

### Activation of OVA-specific naive and effector T cells by mouse DCs

Bone marrow (BM) DCs generated from wt and CD73−/− animals were pulsed with 100 µg/ml OVA or vehicle overnight. DCs were also treated for 30 min with AMP (AMP-OVA-DCs) or vehicle (OVA-DCs) before addition of OVA. DCs (1×10^4^) were collected, washed and co-cultured for 4 days with naive OVA-specific CD4+ T cells (1×10^5^) purified from un-manipulated OTII TCR transgenic animals in round-bottom 96-well tissue culture plates. After 4 days, supernatants were harvested and analyzed for the presence of IFN-γ, IL-5, and IL-13 by ELISA (R&D systems, USA).

### Flow cytometry

Maturation status of DCs was analyzed via flow cytometry. Cells were stained for the expression of the cell surface markers CD40, CD80, CD83 and CD86 (antibodies were from Immunotools, Frisoythe, Germany). In all experiments, dead cells were excluded from analysis using propidium iodide. Analysis was performed on a FacsCalibur flow cytometer, using Cellquest and FlowJo software.

### Statistical Analysis

If not stated otherwise the statistical significance of differences between samples was calculated using ANOVA, followed by Bonferroni comparison test. Differences were considered significant if p<0.05.

## Results

### AMP induces intracellular Ca^2+^ transients in immature dendritic cells

**Figure 1:**
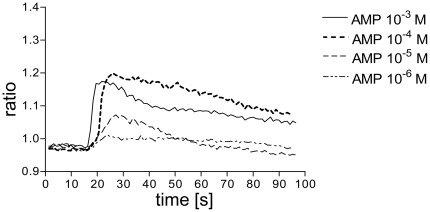
AMP triggers intracellular Ca^2+^ transients in human immature monocyte-derived dendritic cells. Cells were loaded with the Ca^2+^ indicator fura-2/AM and stimulated with the indicated concentrations of AMP. One representative experiment of at least 5 similar is shown.

Intracellular Ca^2+^ transients are crucial for cellular responses such as chemotaxis or cytokine secretion. Stimulation of immature monocyte-derived DCs resulted in a time- and dose- dependent increase in intracellular Ca^2+^ concentration (Fig. 1). Maximal effect was seen at 10^−4^ M AMP, while half-maximal effect was seen at 10^−5^ M.

### AMP stimulates actin polymerization and migration of immature dendritic cells

**Figure 2:**
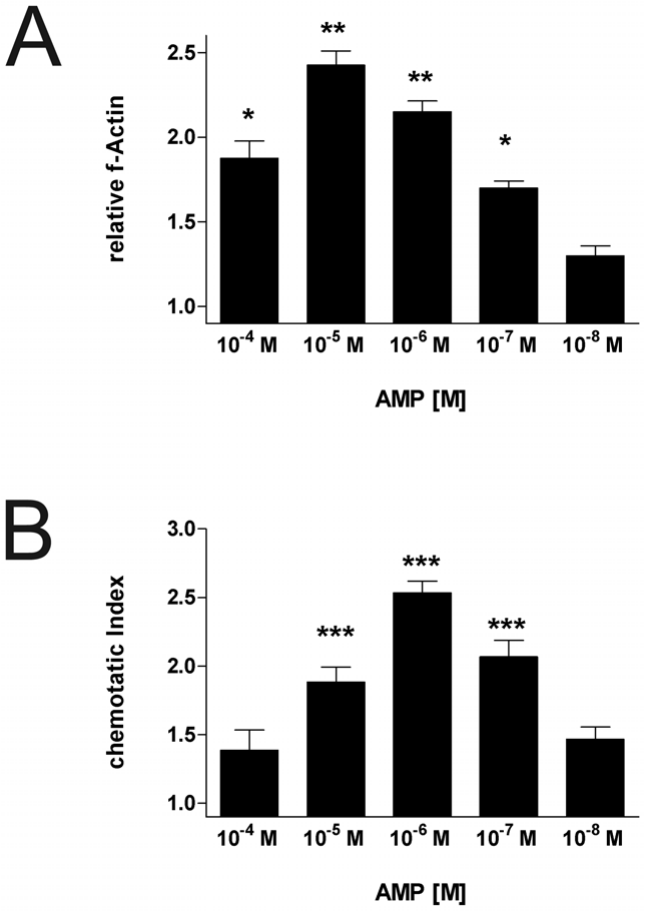
AMP stimulates actin polymerization and migration in immature dendritic cells. Cells were stimulated with the indicated AMP concentrations. The relative f-actin content after 25 s was analyzed (A). Data are means ± SE of 4 independent experiments (*n* = 4). DCs were exposed to the indicated concentrations of AMP for 90 min at 37°C in a Boyden chamber (B). The chemotactic index was calculated. Data are means ± SEM of 4 independent experiments (*n* = 4). * p<0.05 ** p<0.01 *** p<0.001.

Both extracellular nucleotides and adenosine are known chemotactic stimuli for human immature dendritic cells [17], [19]. As polymerization of the intracellular actin network is a prerequisite for oriented migration, the effect of AMP on this process was analyzed. Figure 2A shows that AMP caused a rapid and transient polymerization of the actin network in immature DCs, with an increase in f-actin content of about 50% within 25 seconds. Maximal and half-maximal effects were seen at an AMP concentration of 10^−5^ M and 10^−7^ M, respectively.

Migration of DCs in response to AMP was analyzed using the modified transwell system. As shown in Figure 2B AMP dose-dependently stimulated migration of immature DCs with maximal response at a concentration of 10^−6^ M and half-maximal response at 10^−5^ M. However, AMP had no chemotactic activity on LPS-matured DCs (data not shown).

### AMP regulates cytokine secretion of human dendritic cells

Both extracellular nucleotides and adenosine have been shown to be potent modulators of cytokine secretion. Therefore, the effect of AMP on cytokine release by LPS-matured DCs was analyzed.

**Figure 3:**
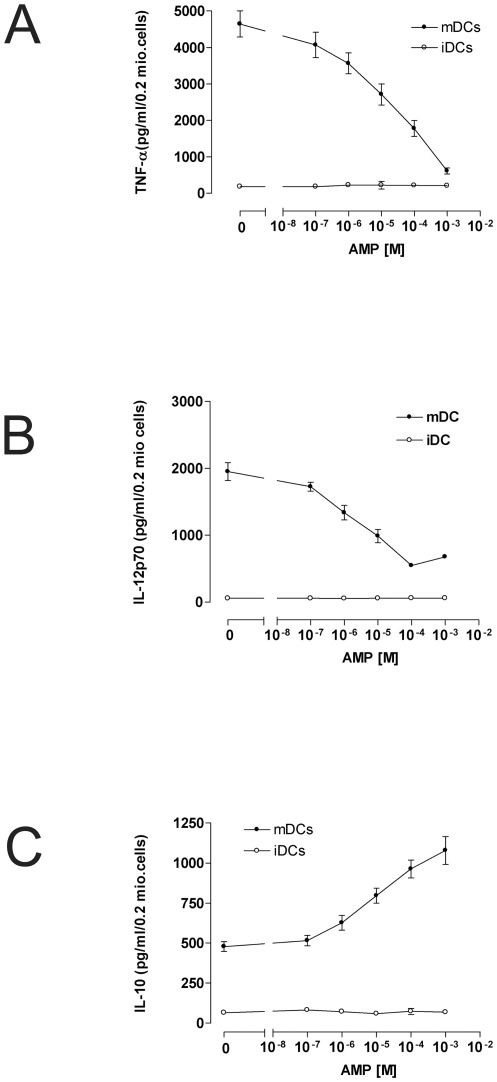
AMP regulates cytokine secretion of human monocyte-derived dendritic cells. 0.2×10^6^ cells were stimulated with the indicated concentrations of AMP. LPS (3 µg/ml) or vehicle was added one hour later. Cells were incubated for 24 h and contents of TNF-α (A), IL-12p70 (B), and IL-10 (C) by unpulsed immature dendritic cells (iDCs) or LPS-pulsed mature dendritic cells (mDCs) were determined by ELISA. Data are means ± SEM of triplicate culture. One representative experiment of at least 5 similar is shown (n = 5). For the average of all 3 experiments see the Supplemental Information (Fig. S1A–C).

As shown in figure 3 AMP inhibited production of the pro-inflammatory cytokines TNF-α and IL-12p70 in a dose dependent manner. Maximal and half-maximal inhibition of TNF-α secretion was seen at an AMP concentration of 10^−3^ M and 10^−5^ M respectively (Fig. 3A, Fig. S1A). Maximal and half-maximal effect on IL-12p70 release was obtained at 10^−4^ and 10^−6^ M (Fig. 3B, Fig. S1B). AMP also modulated production of the regulatory cytokine IL-10: AMP dose-dependently increased IL-10 secretion by LPS-primed DCs. Maximal effect was obtained by 10^−3^ M AMP, while half maximal response was seen at 10^−5^ M (Fig. 3C, Fig. S1C).

Moreover, AMP at a concentration of 10^−4^ M alone or in combination with LPS induced increased cell surface expression of CD83 and CD86 whereas no significant changes could be observed in CD40 or CD80 expression (data not shown).

### AMP affects T-cell priming of human monocyte-derived dendritic cells

As IL-12p70 is the most important cytokine involved in the differentiation of Th1 cells, we analyzed cytokine secretion of T cells induced by DCs primed with AMP. DCs were stimulated with 10^−4^ M AMP in the presence or absence of LPS. After 24 h they were co-cultured for 5 days with allogeneic CD4^+^CD45RA^+^ T-cells. Cytokine secretion of T cells was analyzed by ELISA.

**Figure 4:**
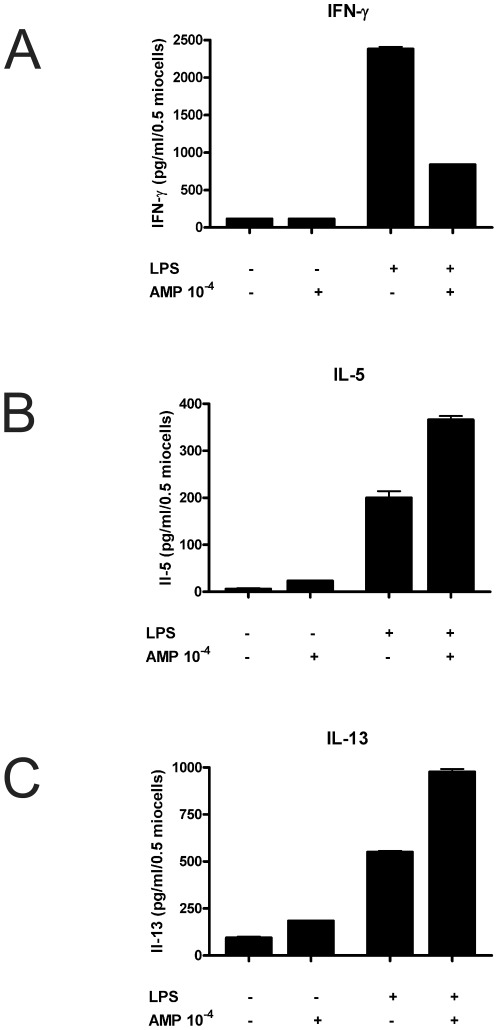
AMP influences T-cell priming capacity of human monocyte-derived dendritic cells. Immature DCs were left untreated or stimulated with 10^−4^ M AMP or induced to undergo maturation with LPS in the absence or the presence of AMP for 24 hours. DCs were then used to prime purified allogeneic CD4^+^CD45RA^+^ naive T-lymphocytes. After 5 days, T cells were restimulated with PMA and ionomycin, supernatants were taken and analyzed for content of IFN-γ (A), IL-5 (B) and IL-13 (C). Data are means ± SEM of triplicate culture. One representative experiment of 3 similar is shown (n = 3). For the average of all 3 experiments see the Supplemental Information (Fig. S2).

As shown in Fig. 4 AMP did not alter IFN-γ, IL-5, or IL-13 secretion induced by immature DCs. However, treatment of DCs with AMP during maturation strongly inhibited IFN-γ release by CD4^+^CD45RA^+^ T-cells, whereas IL-5 and IL-13 secretion was increased (Fig. 4, Fig. S2).

### AMP-induced responses are partially inhibited by adenosine receptor antagonists

Direct activation of adenosine receptors by AMP has been shown previously [6], [7]. Therefore, experiments with different adenosine receptor antagonists were performed. Pretreatment of DCs with the A_1_ receptor antagonist DPCPX completely blocked intracellular Ca^2+^ increases and actin polymerization induced by AMP (Table 1). Chemotaxis was partly inhibited by DPCPX and to a lesser degree by the A_3_ receptor antagonist MRS1220. Modulation of cytokine secretion by AMP was almost completely abrogated by the A_2α_ antagonist ZM241385.

**Table 1:** Characterization of the involved receptors in immature and LPS-differentiated dendritic cells by using selective antagonists.

Cell type/variable	control	AMP	AMP+DPCPX	AMP+ZM241385	AMP+MRS1220
**iDCs**					
**Ca^2+^ transients**	0.88±0.05	1.22±0.06#	1.07±0.04*	1.24±0.03	1.20±0.04
**actin polymerization**	1.00±0.00	1.95±0.18#	1.35±0.10*	1.89±0.12	1.90±0.12
**Chemotaxis**	1.00±0.00	2.10±0.20#	1.30±0.15*	2.15±0.18	1.70±0.12*
**mDCs**					
**TNF- α**	4200±590	1050±280#	1180±450	3950±690*	1200±380
**IL-12p70**	1950±450	590±280#	630±250	1650±390*	570±190
**IL-10**	450±125	1180±220#	980±180	580±175*	930±190

Cells were pre-incubated for 30 min with the A_1_ receptor antagonist DPCPX, the A_2_ receptor antagonist ZM241385, and the A_3_ antagonist MRS1220 at a concentration of 10^−6^ M. Ca^2+^ transients were analyzed, and the ratio after stimulation with AMP at a concentration of 10^−4^ M for 10 s is given. Data are means ± SE (n = 3). Actin polymerization after stimulation with AMP for 25 s was measured and calculated as the ratio between the medium control and stimulated cells. Data are means ± SE (n = 3). The chemotactic index after stimulation with AMP at 10^−5^ M was calculated as the ratio between stimulated cells and cells incubated with medium. Data are means ± SE (n = 3). TNF-α, IL-12p70, and IL-10 release after costimulation with LPS (3 µg/ml) and 10^−4^ M AMP was calculated, and cytokine content is given as pg/ml/0.2×10^6^ cells. Data are means ± SEM (n = 3).

# p<0.05 compared to control,

* p<0.05 compared to AMP-stimulated cells.

### Adenosine independent effect of AMP on cell function of bone marrow derived DC from WT and CD73−/− animals and of human DC

AMP can be hydrolyzed rapidly to adenosine by membrane bound 5′-nucleotidase (CD73). Expression of CD73 on immature and mature human monocyte-derived DCs and on murine bone-marrow derived dendritic cells (BMDCs) has been reported previously [23]. To better define the role of CD73 in AMP induced cell function, we performed experiments with BMDCs generated from wt and CD73−/− animals.

**Figure 5:**
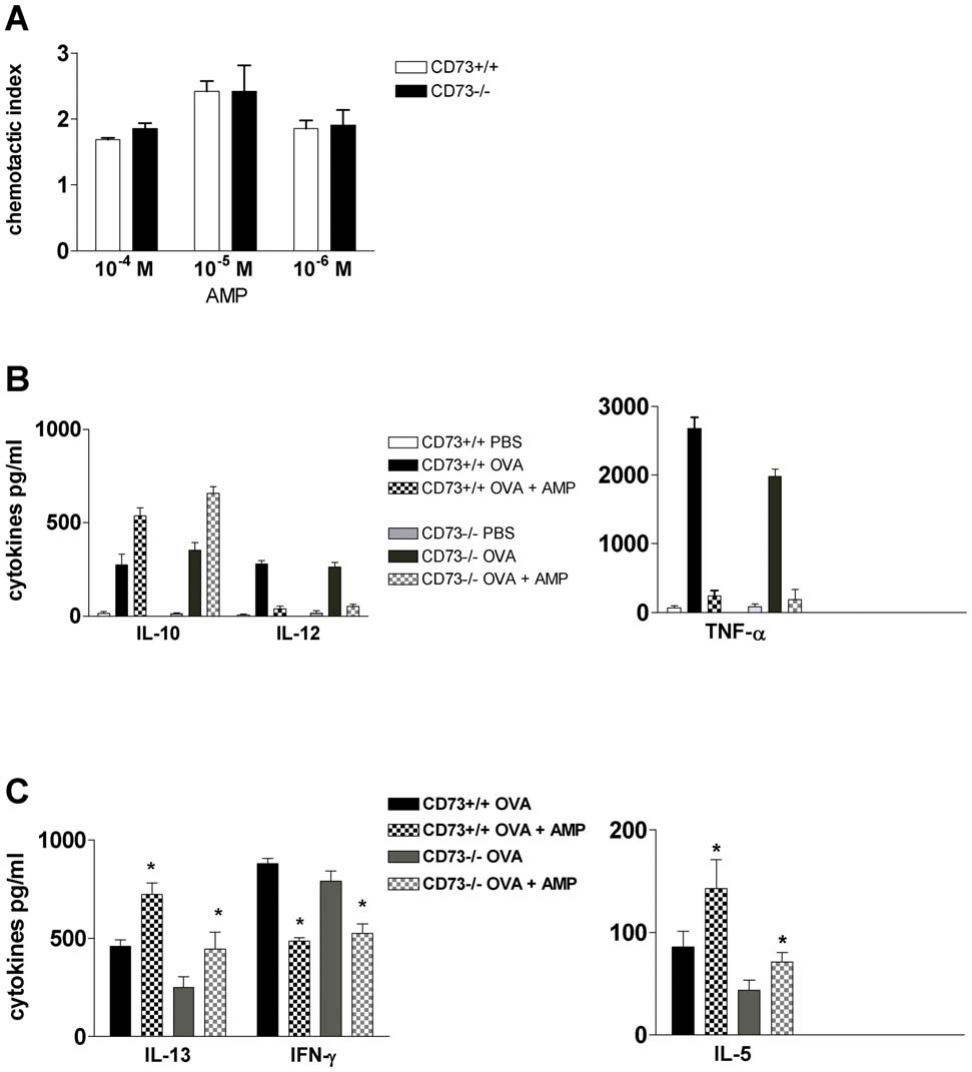
Effect of AMP on CD73+/+ and CD73−/− BMDCs. A) AMP induced migration of immature BMDC generated from CD73+/+ and CD73−/− animals. DCs were stimulated with the indicated concentrations of AMP for 90 min. Results are shown as chemotatic index, calculated as the number of cells in the lower chamber containing the different stimuli divided by the number of cells in the chamber containing medium alone. One representative data out of 3 experiments is shown. B) CD73+/+ and CD73−/− BMDCs were incubated with AMP (10^−4^ M) or vehicle overnight. Supernatants were collected and IL-10, IL-12, TNF-α concentrations were measured in supernatants by ELISA. Data are means ± SEM of triplicate culture. One representative out of 3 experiments is shown. C) T-cell priming. OVA-DC generated from CD73+/+ and CD73−/− animals were stimulated with AMP (10^−4^ M) or vehicle prior to co-culture with OT-II naive T-cells for 5 days in vitro. Levels of IFN-γ, IL-5 and IL-13 were measured in the supernatants. Data are means ± SEM, n = 3. * p<0.05.

BMDCs were stimulated with increasing concentrations of AMP and the chemotactic index was calculated as described above [21], [22]. Interestingly, AMP dose-dependently induced migration in both wt and CD73−/− immature (PBS-pulsed) DCs but not in OVA-pulsed DCs (data not shown), suggesting that AMP-induced migration is independent from the extracellular generation of adenosine (Figure 5A).

BMDCs derived from wt or CD73−/− mice were also stimulated with AMP or vehicle 30 min prior to pulsing with OVA or PBS for 24 h. The next day, levels of IL-12, TNF-α and IL-10 in supernatants were assessed by ELISA. As expected, AMP suppressed IL-12 and TNF-α production, while IL-10 production in OVA-pulsed BMDCs was increased (Figure 5B). Of note, AMP was still able to modulate IL-10, IL-12 and TNF-α release in OVA-pulsed CD73−/− BMDCs, supporting our assumption that AMP can affect DC function at least partially independent of extracellular adenosine generation.

According to our experiments with human monocyte derived DCs, we questioned whether AMP can also modulate T cell priming capacity of BMDCs in vitro. Therefore, BMDCs derived from wt and CD73−/− animals were stimulated with AMP (10^−4^ M) or vehicle 30 min prior to overnight pulsing with OVA or PBS. Cells were then washed twice and co-cultured with purified naïve CD4+ T cells from OTII animals. As shown in Figure 5C, OTII cells which had been co-cultured with AMP-treated OVA-pulsed DCs derived from wt or CD73−/− animals 5 days produced higher levels of the Th2 cytokines IL-5 and IL-13 and lower IFN-γ levels compared to vehicle treated DCs.

**Figure 6:**
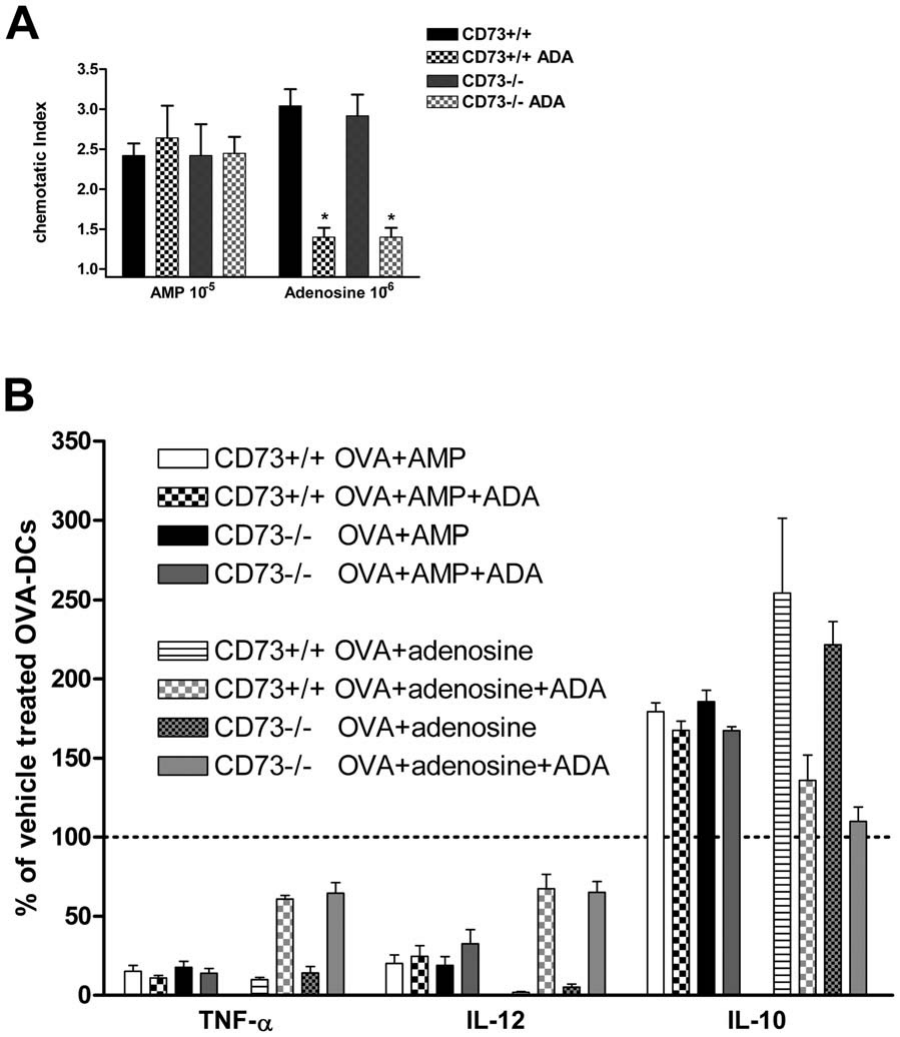
Contaminating adenosine is not involved in AMP induced cell function. A) Adenosine-independent migration of immature BMDC generated CD73+/+ and CD73−/− in response to AMP. DCs were stimulated with AMP (10^−5^ M) or Adenosine (10^−6^ M) in the absence or present of ADA (1 IU/ml) for 90 min. Results are shown as chemotatic index, calculated as the number of cells in the lower chamber containing the different stimuli divided by the number of cells in the chamber containing medium alone. One representative out of 3 experiments is shown (n = 3). B) CD73+/+ and CD73−/− BMDCs were incubated with OVA, adenosine (10^−4^ M) and AMP in the presence or absence of adenosine deaminase/ADA (1 IU/ml) overnight. Supernatants were collected and IL-10, IL-12, TNF-α concentrations were measured in supernatants by ELISA. Data are means ± SEM of triplicate culture. One representative out of 3 experiments is shown (n = 3).

It has been assumed that commercially available AMP could be contaminated with adenosine. Thus to exclude any effects induced by contaminating adenosine in our experiments, AMP and adenosine were ad-mixed with adenosine desaminase prior to stimulation of BMDCs from wt or CD73−/− animals [24], [25], [26]. As shown in Figure 6 (A–B), the administration of 1 IU of adenosine desaminase (ADA) did not significantly influence AMP-induced migration of immature DCs and cytokine secretion of OVA-matured DCs, while it strongly reduced adenosine-induced effects.

In accordance with our results with murine BMDCs, inhibition of CD73 by APCP did influence neither AMP-induced Ca^2+^ transients, nor actin polymerization, nor migration in human monocytes-derived DCs (table 2).

**Table 2:** Influence of the ecto-nucleotidase inhibitor APCP and ADA on AMP-mediated effects.

Cell type/variable	control	control+APCP	AMP	AMP+APCP
**iDCs**				
**Ca^2+^ transients**	0.93±0.05	0.87±0.03	1.24±0.04#	1.23±0.05#
**actin polymerization**	1.00±0.00	1.00±0.00	2.04±0.07#	1.98±0.12#
**Chemotaxis**	1.00±0.00	1.00±0.00	2.30±0.14#	1.75±0.13# *
**mDCs**				
**TNF-α**	2180±480	2320±396	480±350#	1150±290# *
**IL-12p70**	3850±350	4050±440	850±300#	2250±350# *
**IL-10**	390±150	350±210	1300±160#	850±170# *

Cells were pre-incubated for 20 min with the ecto-nucleotidase inhibitor APCP (5′-(alpha,beta-methylene) diphosphate) at a concentration of 10^−6^ M. Ca^2+^ transients were analyzed, and the ratio after stimulation with AMP at a concentration of 10^−4^ M for 10 s is given. Data are means ± SEM (n = 3). Actin polymerization after stimulation with AMP for 25 s was measured and calculated as the ratio between the medium control and stimulated cells. Data are means ± SEM (n = 3). The chemotactic index after stimulation with AMP at 10^−5^ M was calculated as the ratio between stimulated cells and cells incubated with medium. Data are means ± SE (n = 3).

# p<0.05 compared to control,

* p<0.05 compared to AMP-stimulated cells.

## Discussion

The nucleotide AMP has been demonstrated to have a variety of biological effects on different cell types [4]. However, the influence of AMP on human or murine dendritic cells have not been investigated yet.

Stimulation of immature dendritic cells resulted in intracellular Ca^2+^ transients, actin polymerization, and oriented migration. Previous studies were able to demonstrate that AMP can bind directly to A_1_ receptors [6], [7]. Interestingly, A_1_ receptor activation has been linked with intracellular Ca^2+^ transients, actin polymerization, and migration in human monocytes-derived DCs [16], [17]. Pretreatment with the A_1_ antagonist DPCPX fully blocked intracellular Ca^2+^ transients and actin polymerization and partly inhibited migration induced by AMP. Hence the involvement of A_1_ receptors in AMP-induced activation of human immature DCs is likely. Additionally, conversion of AMP to adenosine which can then bind to A_3_ receptors occurring during long term stimulation with AMP might explain why DPCPX only partly blocked migration induced by AMP. In accordance, migration of DCs was also slightly inhibited by the A_3_ antagonist MRS1220. There is good evidence that the concentration of extracellular nucleotides e.g. ATP is elevated under hypoxic conditions or in inflamed tissue [27], [28]. Thus it is likely that the concentration of ATP degradation products such as AMP is increased as well. Similar to ATP, AMP might act as a signal molecule leading DCs towards the site of inflammation [19]. However, during the process of maturation expression of A_1_ receptors by DCs is down-regulated [17] and consequently DCs loose their ability to migrate in response to AMP which might be a prerequisite for departure on the way to secondary lymphoid organs.

Cytokines and chemokines secreted by dendritic cells are crucial for the regulation of immune responses [10]. Here we demonstrate that AMP suppresses the release of the cytokines IL-12p70 and TNF-α, whereas it up-regulates IL-10 secretion by maturing dendritic cells. The effects of AMP on cytokine secretion were fully blocked by the A_2a_ antagonist ZM241385. We and others could show previously that LPS-matured DCs expressed only A_2a_ and not A_2b_ receptors [16], [17], [29] whereas Pacheco and colleagues reported also expression of A_2b_ receptors, though surface expression of A_2b_ receptors on mature DCs was very low in this study [30]. As similar effects on cytokine secretion have been seen in LPS-primed DCs following activation of A_2a_ receptors this effect seems to be mediated by direct binding of AMP to this receptor subtype.

DCs treated with AMP during maturation switched to a low TNF-α and IL-12p70/high IL-10 producing phenotype. To evaluate the functional relevance T cell priming capacity of DCs stimulated with AMP was analyzed. AMP itself did not change T cell priming capacity of DCs. However, human DCs matured in the presence of AMP induced a type 2 immune response with up-regulation of IL-5/IL-13 production and inhibition of IFN-γ release. In line with these findings the Th2-priming capacity of OVA-pulsed murine DCs was enhanced when DCs were pretreated with AMP prior to OVA-pulsing. In summary, our results implicate that AMP limits Th1 and favors Th2 responses similar to previous studies in which ATP or adenosine induced Th2 immunity [16], [20].

A concern when conducting experiments with AMP is the enzymatic breakdown to adenosine by membrane bound ecto-nucleotidases (CD73) [31] expressed on both human and murine DCs [23]. For adenosine has been reported to cause similar effects in human and mouse DCs [16], [17], [32], [33], [34], experiments with DCs generated from CD73−/− and WT animals were performed. Strikingly AMP was still able to modulate migration, cytokine production and T cell priming in DCs derived from CD73−/− animals. In accordance, pretreatment of human DCs with the ecto-nucleotidase inhibitor APCP did not abrogate Ca^2+^ transients, actin polymerization and migration elicited by AMP. In conclusion, these data clearly demonstrate that AMP can modulate DC function independently from extracellular adenosine generation. A direct adenosine-independent effect of AMP on DC function could further be supported by our finding that the co-administration of adenosine desaminase, an enzyme metabolizing extracellular adenosine, did not influence AMP induced cell responses, ruling out a potential contamination of AMP with adenosine.

AMP has been used in bronchoprovocation tests for the diagnosis and monitoring of asthma causing both an early and a late phase asthmatic reaction. Interestingly, the AMP bronchoprovocation test has greater disease specificity for bronchial asthma and reflects the degree of inflammation especially in the peripheral airways better than other tests [35], [36], [37], [38]. Evidence on the mechanism of adenosine and AMP-mediated bronchoconstriction has indicated an extracellular site of action and the stimulation or potentiation of mast cell mediator release [37]. However, our data show that the pro-inflammatory effects of AMP could also be due to activation of dendritic cells which are essential for induction and maintenance of asthmatic airway inflammation [15]. As mentioned above, levels of nucleotides in the extracellular space are elevated under inflammatory conditions. Previous studies were able to demonstrate that nucleotides such as ATP and adenosine are involved in the pathogenesis of inflammatory diseases [21], [22], [28], [39], [40]. Our findings show that AMP, a degradation product of ATP, is also able to favor Th2 immune responses by influencing dendritic cell function. Therefore nucleotide metabolism in the extracellular space might be a crucial point in different inflammatory disorders.

In the past it has been assumed that the biological properties of AMP were exclusively due to extracellular conversion of AMP to adenosine. However, our data suggest that AMP can elicit effects similar to adenosine by direct binding to A_1_ and A_2a_ receptors without being de-phosphorylated before. Therefore, the biological effects of AMP are likely to be mediated by both AMP and adenosine [6], [7], [41].

In summary we were able to demonstrate that AMP is a potent regulator of maturing dendritic cells linked to chemotaxis, altered cytokine secretion, and Th2-polarisation. Therefore AMP, similar to ATP or adenosine, might act as a signaling molecule favoring Th2-responses.

## Supporting Information

Figure S1 **Cytokine production by human monocyte-derived dendritic cells.** 0.2×10^6^ cells were stimulated with the indicated concentrations of AMP. LPS (3 µg/ml) or vehicle was added one hour later. Cells were incubated for 24 h and contents of TNF-α (A), IL-12p70 (B), and IL-10 (C) by LPS-pulsed mature dendritic cells (mDCs) were determined by ELISA. The index was calculated (% of LPS-treated cells). Data are mean +/− SEM for 5 independent experiments (n = 5). * p<0.05 compared to LPS-treated DCs.
(PDF)

Figure S2 **AMP influences T-cell priming capacity of human monocyte-derived dendritic cells.** DCs were induced to undergo maturation with LPS in the absence or the presence of AMP for 24 hours. DCs were then used to prime purified allogeneic CD4^+^CD45RA^+^ naive T-lymphocytes. After 5 days, T cells were restimulated with PMA and ionomycin, supernatants were taken and analyzed for content of IFN-γ, IL-5 and IL-13. The index was calculated (% of LPS-treated cells). Data are mean +/− SEM for 3 independent experiments (n = 3). * p<0.05 compared to LPS-stimulated DCs.
(PDF)
