# Supplementary figures and images for: AMP Affects Intracellular Ca2+ Signaling, Migration, Cytokine Secretion and T Cell Priming Capacity of Dendritic Cells
Source: PLoS One. 2012 May 18;7(5):e37560. doi: 10.1371/journal.pone.0037560 (PMC3356328; doi:10.1371/journal.pone.0037560)

A)

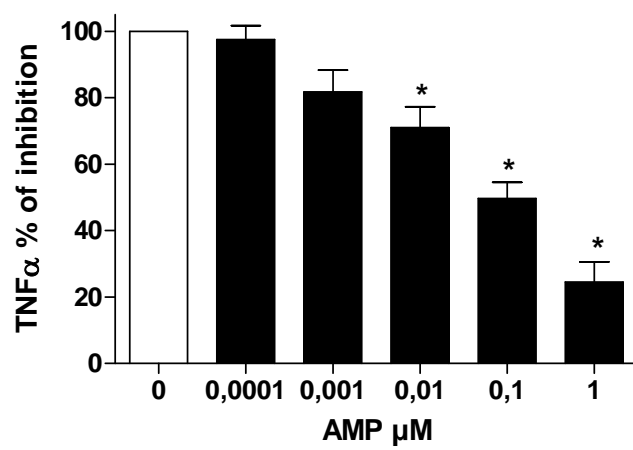

B)

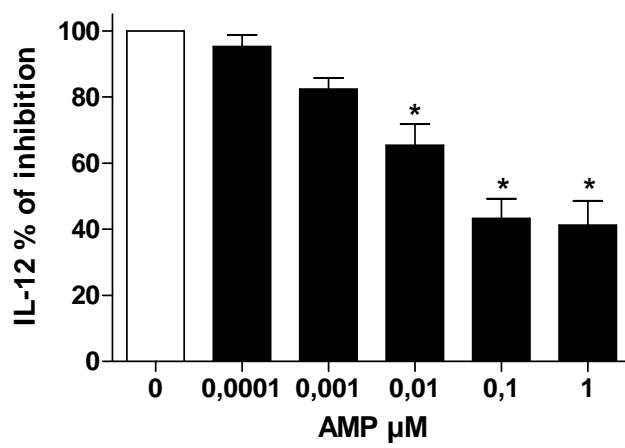

C)

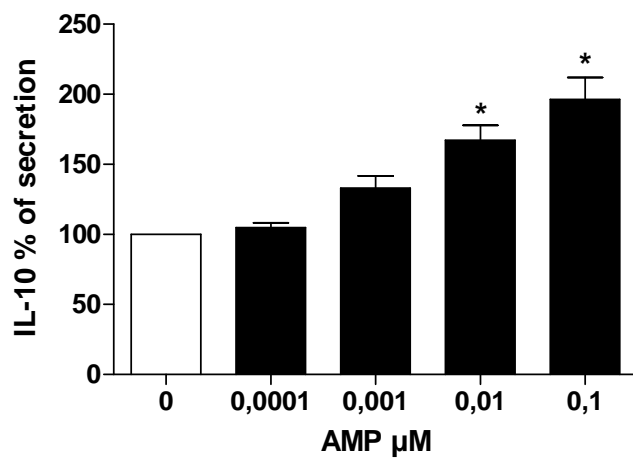

Supplement: Figure S1 — Cytokine production by human monocyte-derived dendritic cells. 0.2×10^6^ cells were stimulated with the indicated concentrations of AMP. LPS (3 µg/ml) or vehicle was added one hour later. Cells were incubated for 24 h and contents of TNF-α (A), IL-12p70 (B), and IL-10 (C) by LPS-pulsed mature dendritic cells (mDCs) were determined by ELISA. The index was calculated (% of LPS-treated cells). Data are mean +/− SEM for 5 independent experiments (n = 5). * p<0.05 compared to LPS-treated DCs. (PDF) [file pone.0037560.s001.pdf]

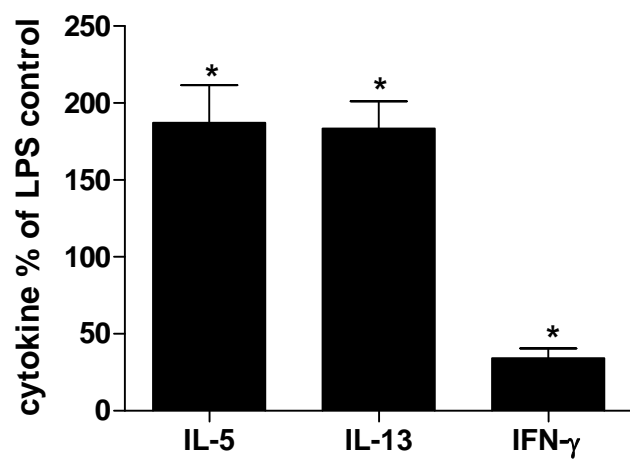

Supplement: Figure S2 — AMP influences T-cell priming capacity of human monocyte-derived dendritic cells. DCs were induced to undergo maturation with LPS in the absence or the presence of AMP for 24 hours. DCs were then used to prime purified allogeneic CD4+CD45RA+ naive T-lymphocytes. After 5 days, T cells were restimulated with PMA and ionomycin, supernatants were taken and analyzed for content of IFN-γ, IL-5 and IL-13. The index was calculated (% of LPS-treated cells). Data are mean +/− SEM for 3 independent experiments (n = 3). * p<0.05 compared to LPS-stimulated DCs. (PDF) [file pone.0037560.s002.pdf]
